# Oxygen-Defect Enhanced Anion Adsorption Energy Toward Super-Rate and Durable Cathode for Ni–Zn Batteries

**DOI:** 10.1007/s40820-021-00699-z

**Published:** 2021-08-05

**Authors:** Jia Yao, Houzhao Wan, Chi Chen, Jie Ji, Nengze Wang, Zhaohan Zheng, Jinxia Duan, Xunying Wang, Guokun Ma, Li Tao, Hanbin Wang, Jun Zhang, Hao Wang

**Affiliations:** 1 Hubei Key Laboratory of Ferro and Piezoelectric Materials and Devices, School of Microelectronics and Faculty of Physics and Electronic Science, Hubei University, Wuhan, 430062 People’s Republic of China; 2 CAS Key Laboratory of Design and Assembly of Functional Nanostructures, and Fujian Provincial Key Laboratory of Nanomaterials, Fujian Institute of Research on the Structure of Matter , Chinese Academy of Sciences, Fuzhou, 350002 People’s Republic of China; 3 Xiamen Key Laboratory of Rare Earth Photoelectric Functional Materials, Xiamen Institute of Rare Earth Materials, Haixi Institute, Chinese Academy of Sciences, Xiamen, 361021 People’s Republic of China

**Keywords:** Ni–Zn battery, Oxygen defect, Nanotube array, CoNiO_2_ nanosheet, Adsorption energy

## Abstract

The alkaline zinc-based batteries with high energy density are becoming a research hotspot. However, the poor cycle stability and low-rate performance limit their wide application. Herein, ultra-thin CoNiO_2_ nanosheet with rich oxygen defects anchored on the vertically arranged Ni nanotube arrays (O_d_-CNO@Ni NTs) is used as a positive material for rechargeable alkaline Ni–Zn batteries. As the highly uniform Ni nanotube arrays provide a fast electron/ion transport path and abundant active sites, the O_d_-CNO@Ni NTs electrode delivers excellent capacity (432.7 mAh g^−1^) and rate capability (218.3 mAh g^−1^ at 60 A g^−1^). Moreover, our O_d_-CNO@Ni NTs//Zn battery is capable of an ultra-long lifespan (93.0% of initial capacity after 5000 cycles), extremely high energy density of 547.5 Wh kg^−1^ and power density of 92.9 kW kg^−1^ (based on the mass of cathode active substance). Meanwhile, the theoretical calculations reveal that the oxygen defects can enhance the interaction between electrode surface and electrolyte ions, contributing to higher capacity. This work opens a reasonable idea for the development of ultra-durable, ultra-fast, and high-energy Ni–Zn battery.

**Supplementary Information:**

The online version contains supplementary material available at 10.1007/s40820-021-00699-z.

## Highlights

- Ultra-thin CoNiO_2_ nanosheet with rich oxygen defects anchored on the vertically arranged Ni nanotube arrays (O_d_-CNO@Ni NTs) is successfully constructed.
- The O_d_-CNO@Ni NTs electrode delivers extraordinary electrochemical performance.
- The theoretical calculations reveal that oxygen defects effectively improve the electrochemical kinetics and the surface electronic state structure of O_d_-CNO @ Ni NTs, thus exhibiting strong OH^ −^ adsorption capacity.

**Supplementary Information:**

The online version contains supplementary material available at 10.1007/s40820-021-00699-z.

## Introduction

With the increasing demand for green and sustainable energy storage, advanced energy storage technology like lithium-ion batteries (LIBs) has attracted extensive attentions [1–3]. However, their applications are seriously hindered caused by the Li dendrite formation and the side reactions, which could cause serious degradation and safety problems [4–6]. As an alternative to LIBs, rechargeable alkaline Zn-based batteries (ZBBs) have attracted more and more attentions with high theoretical capacity (820 mAh g^−1^), low cost, high security, and good ionic conductivity [7, 8].

To date, various alkaline Zn-based cathodes have been developed, such as MnO_2_ [9], Ag-based [10], and Ni, Co-based materials (e.g., Ni(OH)_2_ [11, 12], NiO [13], NiSe_2_ [14], Ni_3_S_2_ [15, 16], Co_3_O_4_ [17–19], Co_3_S_4_ [20], NiCo-DH [21, 22], and NiCo_2_O_4_ [23–25]). While MnO_2_–Zn battery has a low working voltage and weak stability, AgO–Zn battery has low stability, poor overcharge tolerance and high cost. Conversely, Ni–Zn batteries (NZBs) have the advantages of high energy density and high output voltage, reversible oxidation–reduction kinetics of Zn/ZnO, low cost and low toxicity, etc. [26]. However, currently reported cathode materials have low cycle stability due to the self-dissolution of the cathode, the corrosion and dendrite formation of the anode, etc. [27]. Thus, the further development of NZBs is severely restricted.

To address the above limitations, many strategies, including structural design, metal ions doping and surface properties optimization, have been attempted (summarized in Table S1) [28, 29]. Structural design is used to strengthen the electrochemical performance of electrode materials. For example, Chao et al. reported that the NiS@Ni_0.95_Zn_0.05_(OH)_2_ used in NZBs has a long life and fast energy response (18.82 kW kg^−1^, peak power output of 30 s) [30]. Ionic doping could promote the transmission of ions/electrons and show more redox reactions, thereby contributing to electrochemical performance [31]. Surface modification with the introduction of defects and higher conductivity additives, the surface reactivity and reaction kinetics of electrode materials are improved [32]. Lu et al. reported a mesoporous nanostructured Co_3_O_4_ with oxygen defects as the cathode of ZBBs, providing an excellent long-life performance (the capacity does not decrease after 60,000 cycles) [19]. However, the energy density and cycle lifespan of the NZBs are still far from up to standard for practical applications due to the poor conductivity, limited exposure to active sites, and large volume variations for cathode materials. In summary, the exploration of cathode materials with ultra-high capacity, high rate capability and long life is still full of challenges and desirability.

Nickel cobaltate has superior electrochemical activity than oxides of single metal nickel or cobalt due to the electronic transition between the different valence states of the elements and the existence of Co^3+^/Co^2+^ and Ni^3+^/Ni^2+^ redox pairs [33]. Shang et al. prepared porous NiCo_2_O_4_ nanosheets, nanowires and nanoplates as cathode of NZBs, which promoted electron transfer and electrochemical reaction, thereby showing excellent electrochemical performance [24]. However, the volume expansion of CoNiO_2_ (CNO) cathode during the charge–discharge, resulting in poor connection of the electron transmission channel and greatly reduced electrical conductivity, thus making its capacity and high-rate performance far from expected. Herein, we develop an unprecedented composite material that combines electrochemically active structures and defect engineering. Ultra-thin CoNiO_2_ nanosheets with abundant oxygen defects (O_d_-CNO) are introduced in situ on the surface of vertically arranged Ni nanotube arrays (Ni NTs). The density functional theory (DFT) reveals that the introduction of oxygen defects can enhance the adsorption energy of OH^−^, thereby improving the cycle stability of the crystal structure during charge–discharge. Simultaneously, the oxygen defects can effectively modulate the surface electronic structure to promote charge storage. As a consequence, the O_d_-CNO@Ni NTs electrode shows excellent rate performance and high specific capacity. Concretely, the assembled O_d_-CNO@Ni NTs//Zn rechargeable battery provides a capacity of 334.9 mAh g^−1^ and has long cycle life (93.0% retention after 5000 times). In addition, the Ni–Zn battery achieves an energy density of 547.5 Wh kg^−1^ and power density of 92.9 kW kg^−1^. Encouragingly, even after the brutal treatment of hammer and fire, it still shows excellent reliability and safety. This study shows that O_d_-CNO@Ni NTs//Zn has high practical application potential in high-performance ZBBs.

## Experimental

### Material Synthesis

#### Fabrication of Ni NTs@Ni Foam

To obtain regular Ni NTs, the Ni NTs@ZnO array must be synthesized. First, Zn(CH_3_COO)_2_·2H_2_O was dissolved in 100 mL anhydrous methanol (CH_3_OH), and nickel foam was placed in the solution and stirred by ultrasound, and then stored at 200 °C for 2 h. Secondly, 0.1 M Zn(NO_3_)_2_·6H_2_O, 0.1 M HMTA(C_6_H_12_N_4_), 0.1 M ammonia and nickel foam were transferred to a 100 mL Teflon-lined stainless steel, heated at 90 °C for 10 h. Then, Ni films were electrodeposited on ZnO nanorods arrays in 0.2 M NiSO_4_·6H_2_O and 0.01 M NH_4_Cl solutions at − 1.5 mA cm^−2^ for 12 min. After a further immersion in 0.01 M HCl solution for 5–10 min, the prepared sample is cleaned repeatedly with deionized water and dried.

#### ***Fabrication of O***_***d***_***-CNO@Ni NTs, O***_***d***_***-CNO and CNO***

According to our previous report, a cobalt-based organic skeleton (Co-MOF@Ni NTs) supported on Ni NTs was prepared [31]. In a typical experiment, 4 M 2-methylimidazole (C_4_H_6_N_2_) solution was quickly added to 0.05 M Co(NO_3_)_2_·6H_2_O solution, and then the prepared Ni nanotube arrays (Ni NTs) were infiltrated and grown for 4 h. The prepared Co-MOF@Ni NTs were immersed in 16 mM NiSO_4_·6H_2_O solution, react at room temperature for 90 min, wash and dry to obtain NiCo-DH@Ni NTs. To obtain O_d_-CNO@Ni NTs, the prepared sample was heated to 350 °C in the Ar environment for 2 h (heating rate of 2 °C min^−1^). In the absence of Ni NTs, the NiCo-DH sample was heated to 350 °C in Ar atmosphere for 2 h (heating rate of 2 °C min^−1^) to obtain the O_d_-CNO sample. Similarly, the NiCo-DH sample was heated in air to 350 °C and held for 2 h (heating rate of 2 °C min^−1^) to obtain CNO. The sample loading capacity is about 0.8–1.2 mg cm^−2^.

### Material Characterization

The morphology and size were studied by scanning electron microscopy (SEM, JEOL JSM-7100F), transmission electron microscopy (TEM), high-resolution TEM (HRTEM) and selected area electron diffraction (SAED). Elemental analysis and morphology measurements were obtained by energy-dispersive X-ray spectrometer (EDX). The structure and chemical composition were characterized by X-ray diffraction (XRD; Bruker D8 Advance diffractometer), X-ray photoelectron spectrometer (XPS; Thermo Fisher Scientific Escalab 250Xi) and electron paramagnetic resonance (EPR; Bruker EMPplus-10/12). The N_2_ adsorption–desorption isotherms were measured by ASAP 2020 analyzer. The Co ion dissolved amount in the electrolyte was tested by the inductively coupled plasma optical emission spectrometry (ICP − OES).

### Electrochemical Measurements

In this experiment, the electrochemical performance of Ni–Zn alkaline battery in a mixture of 4 M KOH + 1 M K_2_CO_3_ + 2 M KF and saturated ZnO, O_d_-CNO@Ni NTs and commercial zinc were used as cathode and anode, respectively. The Chenhua electrochemical workstation (CHI760E) was used for cyclic voltammetry (CV), galvanostatic charge–discharge (GCD) and electrochemical impedance spectroscopy (EIS) tests. Rate performance and cycle life were measured using the NEWARE battery test system (NEWARE, PR China). In the three-electrode electrochemical test using CHI760E, the nickel foam containing active material was directly prepared as working electrode, and saturated calomel electrode (SCE) was used as the reference electrode and platinum electrode (Pt) was used as the counter electrode.

The energy density E (Wh kg^−1^) and power density P (kW kg^−1^) are obtained by following:

1 $$E = \frac{{I\smallint U{\text{d}}t}}{m}$$

2 $$P = \frac{E}{t}$$

where *I* is the discharge current (mA), *U* is the discharge voltage (V), *t* is the discharge time (h), and *m* is the mass load of the active materials (mg).

### Calculation Methods

The first-principle calculations are performed using VASP code [34], based on density functional theory (DFT) [35, 36]. The CNO slab with (001) surface is chosen as calculation model. The ***a*** and ***b*** axes are 8.94 Å × 8.94 Å, while the ***c*** axes are set to 35 Å to ensure sufficient vacuum to avoid interactions between two cycles. By using the Purdue–Burke–Enzehoff (PBE) exchange–correlation functional, the general gradient approximation (GGA) is used to calculate the exchange–correlation energy [37]. The DFT + U method [38, 39] with strong correlation effects was adopted to describe the localization of Co-3d and Ni-3d electrons. The U–J values of Co and Ni are 3.4 and 6.0 eV, respectively [40]. The influence of van der Waals interactions was estimated, and the optimal commutative van der Waals function DFT-D3 is realized [41]. The cutoff energy of the plane wave was 500 eV, and the 3 × 3 × 1 and 5 × 5 × 1 k-point grids in the Monkhorst Pack [42] sampling scheme were used for geometric optimization and computation of electronic properties, respectively. The convergence condition of energy is 10^−4^ eV, and the structures were relaxed until the force on each atom is less than 0.03 eV Å^−1^. Spin polarization is taken into account in all calculations, and structure mapping and charge density visualization were performed using VESTA [43].

The binding energies *E*_b_ of OH ion on the surfaces of CNO are defined as:

3 $$E_{{\text{b}}} = E_{{\text{CNO + OH}}} - \left( {E_{{{\text{CNO}}}} + E_{{{\text{OH}}}} } \right)$$

4 $$E_{{{\text{OH}}}} = E\left( {{\text{H}}_{{2}} {\text{O}}} \right) - 1/2E\left( {{\text{H}}_{{2}} } \right)$$

here, *E*_CNO+OH_ is the total energy of the CNO slab with an adsorbed OH, *E*_CNO_ is the total energy of pristine CNO slab and *E*_OH_ is the total energy of OH. And *E*(H_2_O) and *E*(H_2_) are the total energy of H_2_O and H_2_ molecule, respectively.

## Results and Discussion

### Morphology and Structure

**Fig. 1:**
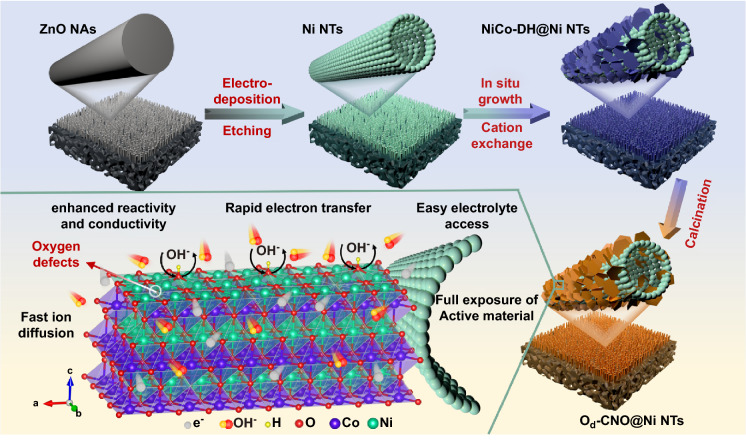
Synthesis mechanism of O_d_-CNO@Ni NTs nanostructure

**Fig. 2:**
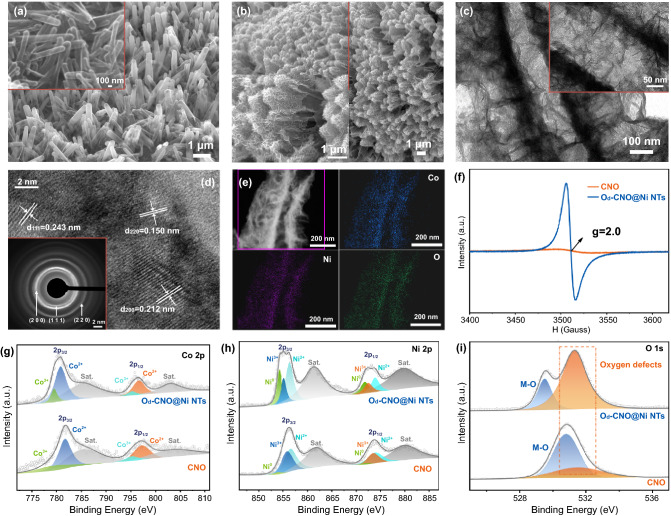
Structure characterization of O_d_-CNO@Ni NTs. **a**, **b** SEM at high and low magnifications of Ni NTs and O_d_-CNO@Ni NTs. **c** TEM images and **d** HRTEM images of O_d_-CNO@Ni NTs. **e** EDX element mappings of O_d_-CNO@Ni NTs. **f** EPR spectra of O_d_-CNO@Ni NTs. **g–i** XPS of Co 2p, Ni 2p, and O 1s for O_d_-CNO@Ni NTs and CNO

Ultra-thin CoNiO_2_ nanosheets with oxygen defects were prepared on nickel nanotubes (O_d_-CNO@Ni NTs) as the cathode material by cation exchange method. The principle of the preparation process is shown in Fig. 1. Firstly, ZnO nanorod arrays (ZnO NAs) were grown on pure nickel foam by hydrothermal method (Fig. S1a–c). The XRD peaks are well retrieved (PDF No. 36-1451, Fig. S1d). Then, uniform Ni films are electrodeposited. ZnO NAs were removed by etching, remaining the Ni NTs with hollow structures (PDF No. 04-0850, Figs. 2a and S2). Finally, the O_d_-CNO nanostructure was prepared by in situ growth on Ni NTs. The Ni NTs are uniformly coated by interconnected ultra-thin O_d_-CNO nanosheets (Fig. 2b), and the size of a single nanosheet was about 200 nm. As a comparison, SEM images of CNO and O_d_-CNO samples are shown in Fig. S3. In addition, the Brunauer–Emmett–Teller (BET) results further demonstrate the high specific surface area of O_d_-CNO@Ni NTs (Fig. S4). Specifically, the specific surface area of O_d_-CNO@Ni NTs (52.25 m^2^ g^−1^) was much higher than that of O_d_-CNO (36.57 m^2^ g^−1^) and CNO (35.84 m^2^ g^−1^). The O_d_-CNO@Ni NTs composite arrays combine the advantages of self-supporting Ni NTs and the abundant active sites of the O_d_-CNO two-dimensional ultra-thin nanosheets, which not only provides more active sites, but also reduces the ion transport distance [44].

To explore the structural differences caused by the introduction of oxygen defects, we also prepared CNO without oxygen defects (Fig. S5). Three diffraction peaks can be assigned to the (111), (200), and (220) crystal faces of CNO (PDF No. 10-0188) [45]. However, the intensity of diffraction peaks of the three main crystal planes of O_d_-CNO is weakened, which means that the crystallinity is weakened to some extent [23]. To further understand the microstructure of the samples, TEM was used to characterize. Figure 2c reveals the diameter of the Ni NTs is approximately 100 nm. The lattice fringes can be seen under HRTEM (Fig. 2d). Concretely, compared with CNO, the lattice fringe spacing of O_d_-CNO has no significant change, which is *d*_(111)_ = 0.243 nm, *d*_(200)_ = 0.211 nm, and *d*_(220)_ = 0.150 nm, respectively. This corresponds to three faintly bright diffractive concentric rings in the SAED pattern (illustration of Fig. 2d), while that of CNO (Fig. S6) are clearer and brighter. As a result, this is consistent with the abovementioned characterization result in Fig. S5. According to the energy-dispersive EDX of O_d_-CNO@Ni NTs in Fig. 2e, Ni, Co, and O are evenly distributed on the nanosheet (Fig. S7 and Table S2). The optical photograph of the composite electrode also shows the uniform distribution of the electrode material on the nickel foam (Fig. S8).

The results of electron paramagnetic resonance (EPR) analysis (Fig. 2f), the oxygen defects characteristic (g factor is a peak signal of 2.0) was generated in O_d_-CNO@Ni NTs lattice [46]. To further prove the existence of oxygen defects and analyze the valence state of each element, the XPS analysis of O_d_-CNO@Ni NTs was carried out. The XPS survey spectrum (Fig. S9) shows Co, Ni, O, and C, which are the main elemental of O_d_-CNO@Ni NTs. Figure 2g demonstrates two typical Co 2p_1/2_ and Co 2p_3/2_ orbitals of the CNO phase. It shows that the Co state exists in the form of Co^2+^ and Co^3+^ [47]. The Ni 2p emission spectra of the CNO and O_d_-CNO@Ni NTs samples (Fig. 2h) show Ni 2p_1/2_ and Ni 2p_3/2_ of spin–orbit doublets [48]. For O_d_-CNO@Ni NTs, the peak intensity of Ni^3+^ is obviously weakened, and the binding energies are 855.2 and 872.9 eV, respectively, while the intensity of Ni^2+^ with binding energies of 856.5 and 873.9 eV is increased, indicating that the reduction of Ni^3+^ to Ni^2+^ is related to sintering and annealing, thus confirming the generation of oxygen defects. In addition to Ni^2+^ and Ni^3+^, Ni^0^ exists in the valence state of Ni, indicating that there are metallic Ni phase spots in O_d_-CNO@Ni NTs. It is easy to understand that the detected Ni^0^ is mainly the presence of elemental nickel in the foamed nickel substrate and part of Ni NTs. Figure 2i is a comparative O 1s XPS spectrum of CNO and O_d_-CNO@Ni NTs samples. The peak intensity (M–O) at 529.8 eV mainly corresponds to the host lattice oxygen in O_d_-CNO@Ni NTs (Co–O/Ni–O). In particular, a more obvious peak intensity appears at 531.3 eV, which is related to the bonding state of the defect O [49].

### Electrochemical Performance of the Electrode

**Fig. 3:**
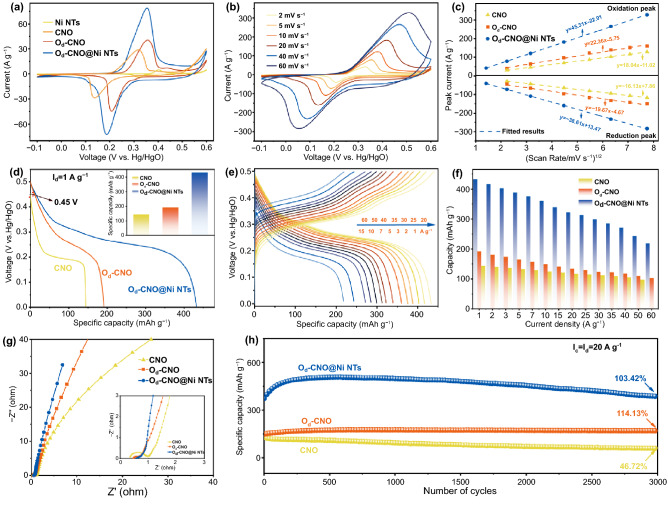
Electrochemical evaluation of the cathodes. **a** Comparison of CV. **b** CV of the O_d_-CNO@Ni NTs at different scan rates. **c** Correlation between peak current and scan rate. **d** Discharge capacity of the O_d_-CNO@Ni NTs, O_d_-CNO and CNO at 1 A g^−1^. **e** GCD of O_d_-CNO@Ni NTs cathode. **f** Rate performance. **g** Nyquist plots. **h** Discharge capacity at 20.0 A g^−1^ for 3000 cycles

The O_d_-CNO@Ni NTs electrochemical performance was characterized in a three-electrode system with 4 M KOH. Figure 3a shows the CV of Ni NTs, CNO, O_d_-CNO, and O_d_-CNO@Ni NTs were collected at 5.0 mV s^−1^ (− 0.1 ~ 0.6 V). The O_d_-CNO@Ni NTs electrode has a larger CV scanning area and higher response current (4.8 A g^−1^ of Ni NTs, 30.2 A g^−1^ of CNO, 40.5 A g^−1^ of O_d_-CNO, 78.8 A g^−1^ of O_d_-CNO@Ni NTs), which confirms its higher capacity storage and electrochemical activity. This may be associated with the promotion of surface charge state and the enhancement of OH^−^ adsorption energy caused by oxygen defects. Besides, compared with CNO, the potential difference of cathodic peak and anodic peak of O_d_-CNO and O_d_-CNO@Ni NTs is much smaller, implying a lower electrochemical polarization for O_d_-CNO or O_d_-CNO@Ni NTs. The CV area of the Ni NTs electrode is quite small, which indicates that the Ni NTs electrode has almost no capacity contribution. CV curves for O_d_-CNO@Ni NTs electrodes from 2 to 60 mV s^−1^ (Fig. 3b), their good symmetrical distribution and similar shape indicate the stability and reversibility of the electrode. The extremely high current density of O_d_-CNO@Ni NTs electrodes indicates its excellent high-power potential.

To study the electrochemical kinetics of O_d_-CNO@Ni NTs electrodes, a typical couple of redox peaks match the adsorption/desorption process of OH^−^ in the redox reaction. The specific reaction equation can be described as:

5 $${\text{CoNiO}}_{2} + 2{\text{OH}}^{ - } { \leftrightharpoons }{\text{CoOOH}} + {\text{NiOOH}} + 2{\text{e}}^{ - }$$

6 $${\text{CoOOH}} + {\text{OH}}^{ - } { \leftrightharpoons }{\text{CoO}}_{2} + {\text{H}}_{{2}} {\text{O}} + {\text{e}}^{ - }$$

The diffusion-controlled redox reaction is revealed by the relation between the peak current density and scan rate (v^1/2^) is linear (Fig. 3c) [14]. Figure S10a–c shows the calculated contribution ratio of the three electrodes at various scan rates. For the O_d_-CNO@Ni NTs electrode, 82% of the capacity is diffusive-controlled at 5 mV s^−1^ and gradually decreases to 51% at 40 mV s^−1^, exhibiting the main diffusive-controlled behavior. For O_d_-CNO electrode and CNO electrode, the capacitance contribution is more obvious. Therefore, the capacity decreases less as the current density increases, benefiting from the surface-control characteristics [33]. Electrochemical impedance spectroscopy (EIS) results also shed light on the enhanced electrochemical kinetics described above. Observed from the Nyquist plot (Fig. 3g), the corresponding equivalent circuit and its values are shown in Fig. S11 and Table S3. In the high-frequency region, a smaller semicircle (inset in Fig. 3g) is shown for O_d_-CNO and O_d_-CNO@Ni NTs electrodes, and their charge transfer resistance *R*_ct_ (0.40 Ω of O_d_-CNO, 0.35 Ω of O_d_-CNO@Ni NTs) is almost half of the CNO electrode (0.72 Ω of CNO). Furthermore, the slope of O_d_-CNO and the O_d_-CNO@Ni NTs is higher in the low frequency region, attributing to oxygen defects introduced in CNO to promote rapid charge transfer, improving the electrode conductivity during charging and discharging. Therefore, the O_d_-CNO@Ni NTs electrode has an overwhelming advantage in terms of electrochemical performance.

Figure 3d shows the comparison of discharge behavior of CNO, O_d_-CNO, and O_d_-CNO@Ni NTs (at 1 A g^−1^). Compared with the CNO (0.45 V), O_d_-CNO and O_d_-CNO@Ni NTs electrodes (0.5 V) have a larger potential voltage window (Fig. S12a–d). The O_d_-CNO@Ni NTs electrode has a more ideal discharge potential platform. The specific capacity of O_d_-CNO@Ni NTs electrodes is as high as 432.7 mAh g^−1^ (at 1 A g^−1^), significantly larger than that of O_d_-CNO electrodes (191.8 mAh g^−1^) and CNO electrodes (144.0 mAh g^−1^). The GCD curves also indicate excellent charge storage capacity (Fig. 3e). The specific capacity reached 432.7, 416.4, 402.6, 387.7, 375.2, 360, 339.2, 322.3, 312.5, 299.2, 284.9, 271.1, 243, and 218.3 mAh g^−1^ at 1, 2, 3, 5, 7, 10, 15, 20, 25, 30, 35, 40, 50 and 60 A g^−1^. The capacity of O_d_-CNO@Ni NTs electrode still retains 218.3 mAh g^−1^ at 60 A g^−1^, demonstrating an impressive rate performance. Comparing O_d_-CNO and CNO electrodes (Figs. 3f and S13a–b), the capacity of O_d_-CNO@Ni NTs electrodes is more than twice that of them, which once again proves the significant effect of hollow Ni NTs on increasing the specific capacity. Furthermore, to solve the main bottlenecks hindering the practical application of alkaline ZBBs, the cycle stability of the CNO, O_d_-CNO, and O_d_-CNO@Ni NTs electrode was evaluated (Fig. 3h). The O_d_-CNO and O_d_-CNO@Ni NTs electrodes showed impressive cycle capacity and structural stability after 3000 cycles (the capacity retention of the O_d_-CNO and O_d_-CNO@Ni NTs electrode were 114.1% and 103.4%, respectively), whereas the capacity retention of CNO electrodes is only 46.7% after 3000 cycles. This serious capacity degradation is related to the deactivation and self-dissolution of the electrode material itself. Importantly, the inductively coupled plasma optical emission spectrometry (ICP-OES) analysis of the Co concentration in electrolyte shows that the most Co ions were dissolved in CNO after 2000 cycles (Fig. S14). Figure S15 shows the SEM of O_d_-CNO@Ni NTs, O_d_-CNO and CNO electrodes at 100, 500, and 2000 cycles, respectively. After the long-cycle of different cycles, the morphology of CNO has obvious changes of dissolution, which eventually leads to the collapse of the structure and sharp attenuation of the capacity (Figs. S15c and S16a–b). However, the morphology of the O_d_-CNO@Ni NTs and O_d_-CNO electrode has no obvious change; thus, the structure is stable. It can still be observed that the clear and orderly hollow tubular structure and ultra-thin nanosheets are coated on the Ni NTs. This once again confirms that oxygen defects can significantly enhance the stability of the material, and abundant nickel nanotubes increase the capacity.

**Fig. 4:**
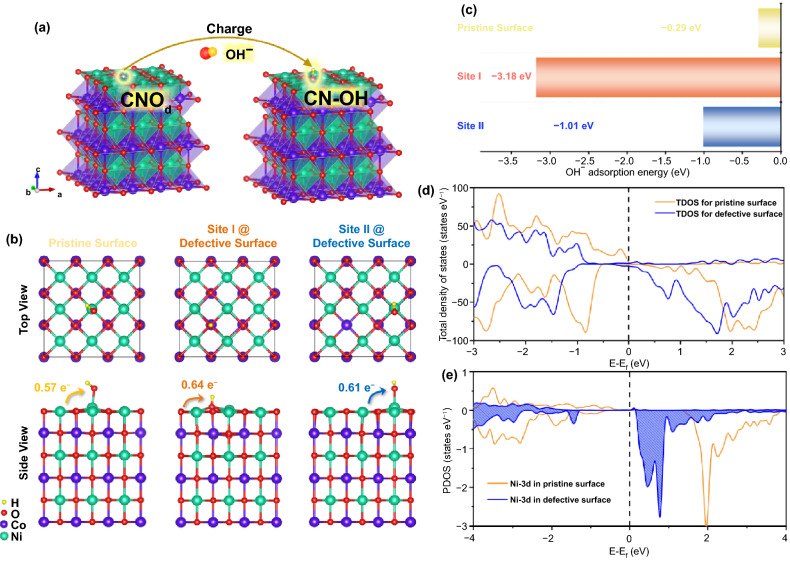
**a** Modulation mechanism model of oxygen defects on OH^−^ adsorption. **b** Surface charge transfer and OH^−^ adsorption energy analysis. **c** Adsorption energy of OH^−^ on CNO and O_d_-CNO. **d** TDOS for CNO and O_d_-CNO. **e** PDOS for CNO and O_d_-CNO

DFT claculation is used to study the impact of oxygen defects on the structure and electronic property. Based on the optimized O_d_-CNO model, the adsorption behavior of OH^−^ was investigated, and the effects of electrochemical performances caused by defects were discussed (Fig. 4a). Two different OH^−^ adsorption sites on the defective surface were considered as shown in Fig. 4b. Apparently, compared with the pristine CNO as shown in Fig. 4c, the introduction of oxygen defects (point defect type/main lattice oxygen defect) can enhance the adsorption of OH^−^ with the decreased binding energies from − 0.29 to − 3.18 or − 1.01 eV, respectively, contributing to higher capacity and cycling stability of the electrode material. This is consistent with the above experimental analysis on the electrochemical performance. Meanwhile, from Bader analysis (Fig. 4b) [50], we found that the existence of oxygen defects could increase the charge transfer between electrode and OH^−^ from 0.57 to 0.64 or 0.61 e^−^, respectively, accounting for the improved adsorption of OH^−^ by oxygen defects.

To further discuss the charge storage mechanism, the total density of states (TDOS) of CNO bulk (Fig. S17), the TDOS of the pristine and O_d_-CNO surface (Fig. 4d), and the partial density of states (PDOS) of the Ni-3d orbits (Fig. 4e) are calculated. From the TDOS of CNO bulk, CNO material has a metallic property, benefit for the charge transport during the electrode reactions. As the surface is the main place for redox reactions, the electronic properties of O_d_-CNO surface are discussed. It can be seen that the empty band above the Fermi energy level is mainly contributed by Ni-3d orbits. After the generation of oxygen defects, the Fermi energy level shifts to a higher energy level, resulting in more empty states near the Fermi energy level. Therefore, these empty states could store more charges, leading to a higher capacity. The intermediate adsorption behavior of OH^−^ on the surface of the active material is very important for the reversible capacity of the electrode material [51]. With the combination of more available unoccupied states and the strong OH^−^ adsorption capacity, the Faraday reversible redox reaction will be promoted, thereby improving the charge storage capacity [52]. The theoretical calculation agrees with our experimental results, indicating that oxygen defects can effectively modify the surface electronic structure and improve the binding energy, rendering faster kinetics and better electrochemical performance.

### Evaluation of the O_d_-CNO@Ni NTs//Zn Aqueous Battery

We use the O_d_-CNO@Ni NTs and zinc foil as cathode and counter electrode, the actual performance of the battery was evaluated in 4 M KOH + 2 M KF + 1 M K_2_CO_3_ + Sat. ZnO electrolyte. To explore the energy storage mechanism of O_d_-CNO@Ni NTs//Zn battery, we conducted a series of ex situ tests, such as ex situ SEM (Fig. S18), ex situ XRD (Fig. S19), ex situ XPS (Fig. S20), and ex situ TEM (Fig. S21). The mechanism of the battery can be understood as following equation [19, 22–25]:

Cathode:

7 $${\text{CoNiO}}_{2} + 3{\text{OH}}^{ - } { \leftrightharpoons }{\text{CoO}}_{2} + {\text{NiOOH}} + {\text{H}}_{{2}} {\text{O}} + 3{\text{e}}^{ - }$$

Anode:

8 $$\left[ {{\text{Zn}}\left( {{\text{OH}}} \right)_{4} } \right]^{2 - } + 2{\text{e}}^{ - } { \leftrightharpoons }{\text{Zn}} + 4{\text{OH}}^{ - }$$

Overall:

9 $$2{\text{CoNiO}}_{2} + 3\left[ {{\text{Zn}}\left( {{\text{OH}}} \right)_{{4}} } \right]^{2 - } { \leftrightharpoons }2{\text{NiOOH}} + 2{\text{CoO}}_{2} + 3{\text{Zn}} + 2{\text{H}}_{{2}} {\text{O}} + 6{\text{OH}}^{ - }$$

**Fig. 5:**
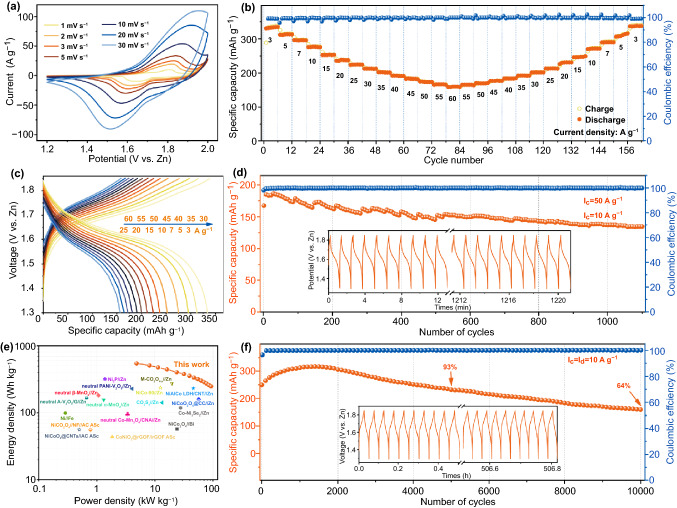
Electrochemical performance of the battery. **a** CV profiles. **b** Rate performance and coulombic efficiency. **c** GCD curves. **d** Cyclic performance and coulomb efficiency at fast charge and slow discharge. The illustration displays the time*–*voltage curves at the first and last 10 cycles. **e** Ragone plot comparing the performance values of the O_d_-CNO@Ni NTs//Zn battery with those of other ZBBs. **f** Cycling performance of the Ni//Zn battery. The illustration displays the time*–*voltage curves at the first and last 10 cycles

Figure 5a illustrates the CV of 1–30 mV s^−1^ (1.2–2 V) for O_d_-CNO@Ni NTs//Zn aqueous battery. At 1 mV s^−1^, the O_d_-CNO@Ni NTs//Zn battery exhibits good symmetrical redox peaks (1.81/1.64 V). Even at 30 mV s^−1^, it still remains symmetric (1.96/1.52 V), which means that the battery has excellent reversibility. Meanwhile, the form of the CV curve remained almost does not change at different scanning rates (1–30 mV s^−1^), proving the battery has excellent stability. Figure 5b shows the battery’s rate capability and coulomb efficiency. The extremely high coulombic efficiency indicates that the battery is ability of rapid charge transfer and ion diffusion. Importantly, the O_d_-CNO@Ni NTs//Zn battery exhibits high capacity of 334.9, 313.4, 296.3, 277.0, 253.3, 238.3, 225.0, 212.5, 202.2, 192.2, 183.8, 175.0, 166.5, and 160.0 mAh g^−1^ when at 3, 5, 7, 10, 15, 20, 25, 30, 35, 40, 45, 50, 55, and 60 A g^−1^, respectively. The average discharge capacity can be restored when switching to 3 A g^−1^ after 156 cycles, which indicates that O_d_-CNO@Ni NTs//Zn battery has an extraordinary rate and reversible stability. Figure 5c shows the GCD curve of the O_d_-CNO@Ni NTs//Zn battery at different current density, showing a flat output voltage (1.62 V). The O_d_-CNO@Ni NTs//Zn aqueous battery can be effectively charged/discharged in about 9.5 s at an extremely high current density (Fig. S22). It still maintains a high capacity of 158.3 mAh g^−1^ (at 60 A g^−1^), implying its ultra-fast properties, providing the possibility of achieving fast charging. Further, Fig. 5d is a graph of the cycle performance of the O_d_-CNO@Ni NTs//Zn battery under fast charge (50 A g^−1^) and slow discharge (10 A g^−1^) conditions. After 1100 cycles (approximately 20.3 h), the capacity retention still exceeded 80%. Comparing the GCD curve before and after the cycle (inset in Fig. 5d), there is no obvious change before and after the cycle, indicating the excellent stability of O_d_-CNO@Ni NTs//Zn battery under fast charge and slow discharge. It again demonstrates that hollow nickel nanotubes and abundant oxygen defects enhance the stability of the material during rapid charging and slow discharge repeatedly. Such excellent performance is almost unreported in current ZBBs.

An ultra-long cycle is realized in the O_d_-CNO@Ni NTs//Zn battery (Fig. 5f). After multiple electrochemical activations, the coulombic efficiency remains nearly 100%. Moreover, as calculated, after 5000 cycles, the O_d_-CNO@Ni NTs//Zn battery at 10 A g^−1^ with a capacity of 232.7 mAh g^−1^, which is 93% of the initial average capacity. After 10,000 cycles (approximately 22 days), the cycle stability reached 64%. Furthermore, the Ragone plot compares O_d_-CNO@Ni NTs//Zn battery with the most advanced aqueous electrochemical system. Encouragingly, in Fig. 5e, our work proposes a maximum energy density is 547.5 Wh kg^−1^ (based on the mass of the O_d_-CNO@Ni NTs cathode), and a maximum power density is 92.9 kW kg^−1^. This performance is better than almost all reported aqueous ZBBs, including, alkaline Ni–Zn [53], Co–Zn [19], Ni–Bi [54], Ni–Fe batteries [55], neutral Zn–Mn [56], Zn–V batteries [57]. Besides, we noticed that supercapacitors also use nickel and cobalt bases as cathodes. In contrast, the energy density of supercapacitors is very low [58].

### Evaluation of the O_d_-CNO@Ni NTs//Zn Soft-Pack Battery

**Fig. 6:**
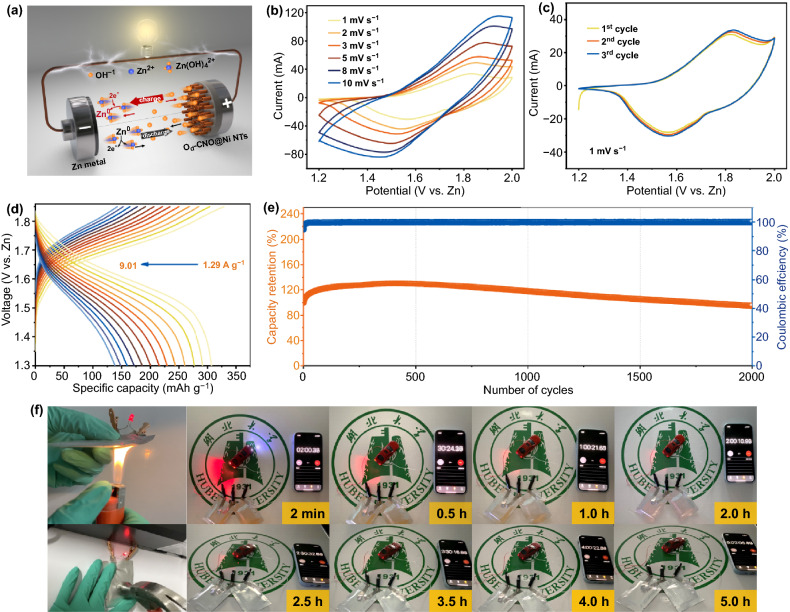
**a** Schematic illustration of the O_d_-CNO@Ni NTs//Zn battery. The O_d_-CNO@Ni NTs//Zn soft-pack battery **b** CV profiles. **c** CV curves for the first three cycles at 1 mV s^−1^. **d** GCD profiles. **e** Cycle stability at 5 A g^−1^. **f** Photograph of safety tests and powering a 3 V model car

Finally, in order to verify the possibility of application in real life, a soft-pack O_d_-CNO@Ni NTs//Zn battery was prepared. The configuration illustration of soft-pack battery is shown in Fig. 6a. As shown in the CV curves in Fig. 6b, c and S23, the redox peak represents the electrochemical process. The soft-pack battery is also capable of achieving high rate performance and long cycle life. At the current density of 1.29, 1.93, 2.58, 3.22, 3.86, 4.51, 5.15, 5.79, 6.44, 7.08, 7.73, 8.37, and 9.01 A g^−1^, the high capacity of 307.22, 291.84, 276.25, 260.19, 243.56, 229.07, 216.02, 199.89, 185.44, 171.72, 159.44, 148.08, and 138.19 mAh g^−1^, respectively (Fig. 6d). The capacity retention is still above 90% after 2000 cycles at 5 A g^−1^ (Fig. 6e). As a result, 3 V car lights are selected as the load for the two series soft-pack batteries. The car lights are very bright and can be kept on for more than 5 h (Fig. 6f). More importantly, comparing the CV before and after exposure to fire and hammer (Fig. S24a–b), the soft-pack battery can work continuously and stably under fire and hammer test (Fig. 6f, Videos S1 and S2). No danger of fire and blast, showing excellent reliability and security.

## Conclusion

In summary, this work developed a three-dimensional hierarchical structure with ordered vertical nanotubes arrays and defective nanosheets, which greatly enhanced the electrochemical performance. Specifically, the existence of Ni NTs can increase ion diffusion channels and shorten ion migration distance, thereby having high conductivity and abundant active sites. More importantly, oxygen defects effectively improve the electrochemical kinetics of the cathode, make the electrode maintain good reversibility for a long time, and improve the surface electronic state structure of O_d_-CNO @ Ni NTs, thus exhibiting strong OH^−^ adsorption capacity. As a result, the O_d_-CNO@Ni NTs cathode shows improved specific capacity (432.7 mAh g^−1^) and extraordinary rate performance (218.3 mAh g^−1^ at 60 A g^−1^). The capacity of the prepared O_d_-CNO@Ni NTs//Zn rechargeable battery is 334.9 mAh g^−1^, and the cycling stability is 93%. At the same time, it still has a capacity retention of 80% under the condition of fast charge (50 A g^−1^) and slow discharge (10 A g^−1^) after 1100 cycles. Our battery can also work at high temperature and high pressure, which will bring immediate benefits to the development for next-generation high-safety commercial batteries.

## Supplementary Information

Below is the link to the electronic supplementary material.

Supplementary file1 (PDF 10297 kb)

Supplementary file2 (MP4 657 kb)

Supplementary file3 (MP4 11718 kb)
