# Supplementary material for: Oxygen-Defect Enhanced Anion Adsorption Energy Toward Super-Rate and Durable Cathode for Ni–Zn Batteries
Source: Nanomicro Lett. 2021 Aug 5;13:167. doi: 10.1007/s40820-021-00699-z (PMC8342634; doi:10.1007/s40820-021-00699-z)
Supplement: Supplementary file 1 — Supplementary file1 (PDF 10297 kb) [file 40820_2021_699_MOESM1_ESM.pdf]

Oxygen-Defect Enhanced Anion Adsorption Energy toward Super-Rate and Durable Cathode for Ni-Zn Batteries

Jia Yao[1], Houzhao Wan[1, *], Chi Chen[2, 3, *], Jie Ji[1], Nengze Wang[1], Zhaohan Zheng[1], Jinxia Duan[1], Xunying Wang[1], Guokun Ma[1], Li Tao[1], Hanbin Wang[1], Jun Zhang[1], Hao Wang[1, *]

[1]Hubei Key Laboratory of Ferro & Piezoelectric Materials and Devices, School of Microelectronics and Faculty of Physics and Electronic Science, Hubei University, Wuhan 430062, China.

[2]CAS Key Laboratory of Design and Assembly of Functional Nanostructures, and Fujian Provincial Key Laboratory of Nanomaterials, Fujian Institute of Research on the Structure of Matter, Chinese Academy of Sciences, Fuzhou, 350002, China.

[3] Xiamen Key Laboratory of Rare Earth Photoelectric Functional Materials, Xiamen Institute of Rare Earth Materials, Haixi Institute, Chinese Academy of Sciences, Xiamen 361021, China.

*Corresponding author. E-mail: Houzhao Wan, houzhaow@hubu.edu.cn; Chi Chen, xmchenchi@fjirsm.ac.cn; Hao Wang, wangh@hubu.edu.cn, nanoguy@126.com

Table 1 Comparison of the electrochemical performance of alkaline Zn-based batteries.

Cathode//anode materials	Electrolyte	$E_{mid,d}$ /V	Specific capacity /mAh g^{-1}	$W_{M,max}$ /Wh kg^{-1}	$P_{M,max}$ /kW kg^{-1}	Cycling life /%	Ref.
O_d-CNO@Ni NTs//Zn foil	4 M KOH + 2 M KF + 1 M K_2CO_3 + sat. ZnO	1.62	338(3.0 A g^{-1})	547.48	92.9(248)	93.0 $_{5000\ cycles}$	This work
Ni–NiO/CC//Zn	6 M KOH + 0.5 M $ZnAc_2$	1.75	256(0.625 A g^{-1})	441.7	41.6(287)	87.5 $_{2000\ cycles}$	[1]
P-$NiCo_2O_{4-x}$//Zn foil	1 M KOH + 50 mM $ZnAc_2$	1.71	366(3.0 A g^{-1})	616.5	30.2 (388)	71.4 $_{5000\ cycles}$	[2]
Co_3S_4//Zn foil	3 M KOH + 0.1 M $ZnAc_2$	1.70	317(1.0 A g^{-1})	366.1	11.8(141)	82 $_{5000\ cycles}$	[3]
NiS-coated $Ni_{0.95}Zn_{0.05}(OH)_2$//Zn mesh	4 M KOH + 2 M KF +1 M K_2CO_3 + sat. ZnO	1.71	360(1mA cm^{-2})	299.6	5.9(236.8)	79.03 $_{500\ cycles}$	[4]
Ni_3S_2@PANI//Zn plate	6 M KOH + 0.2 M $ZnAc_2$	1.71	242(5.1 A g^{-1})	386.7	38.8 (194)	100 $_{5000\ cycles}$	[5]
$NiCo_2O_4$//Zn plate	6 M KOH + 0.1 M $ZnAc_2$	1.70	212(2.0 A g^{-1})	301.5	13.2 (226)	63.2 $_{1000\ cycles}$	[6]
Ni_2P//Zn@CF	1 M KOH + 20 mM $ZnAc_2$	1.78	231(1.0 A g^{-1})	318	-	80.0 $_{1500\ cycles}$	[7]
M-Co_3O_{4-x}//Zn foil	1 M KOH + 50 mM $ZnAc_2$	1.72	288(4.0 A g^{-1})	772.4	17.8 (265)	100 $_{60000\ cycles}$	[8]
$NiCo_2O_4$@CC//Zn@CC	1 M KOH + 20 mM $ZnAc_2$	1.71	183 (1.6 A g^{-1})	303.8	49.0 (159)	82.7 $_{3500\ cycles}$	[9]
NiCo-90//Zn foil	2.5M KOH + sat. ZnO	1.66	299(2.0 A g^{-1})	391.7	11.1 (235)	73.0 $_{850\ cycles}$	[10]

Co-Ni_3Se_2//Zn foil	1 M KOH	1.72	152 (3.0 A g^{-1})	199.3	24.5 (118)	77.9 $_{100\ cycles}$	[11]
NCS@NCH//Zn	2 M KOH + 0.02 M $ZnAc_2$	1.60	135(0.5 A g^{-1})	944.8	14.0(77.7)	105.1$_{4000\ cycles}$	[12]
FNCP//Zn	1 M KOH	1.65	318(1.0 A g^{-1})	532.7	18(146)	90.6 $_{2000\ cycles}$	[13]
Al-CoNiDH-5%//Zn	2.5 M KOH + sat. ZnO	1.59	264(0.5mA cm^{-2})	-	-	64.4 $_{2000\ cycles}$	[14]
Ni_3S_2/O_v-$Ni(OH)_2$//Zn	1 M KOH + 20 mM $ZnAc_2$	1.71	222 (1.0 A g^{-1})	384.6	1.73(275)	93.2 $_{3000\ cycles}$	[15]
CNMO-15//Zn	1 M KOH + 10 mM $ZnAc_2$	1.77	271(2.0 A g^{-1})	474.1	10.3(118.8)	100 $_{5000\ cycles}$	[16]
NiAlCo-LDH/CNT//Zn @Cu foil	1 M KOH + 50 mM $ZnAc_2$	1.75	278(66.7 A g^{-1})	324	40.0 (225)	85.0 $_{600\ cycles}$	[17]
β-$Ni(OH)_2$/CNFs//Zn foil	6 M KOH +1 M LiOH + PAAS + sat. ZnO	1.80	184(5 mA cm^{-2})	325	11.4 (166)	96.0 $_{1200\ cycles}$	[18]
Ni_3S_2@NF//Zn foil	1 M KOH + 20 mM $ZnAc_2$	1.78	68(5.0 A g^{-1})	223.3	7.3 (109)	100 $_{100\ cycles}$	[19]
$NiSe_2$//Zn foil	6M KOH + sat. ZnO	1.74	244(1.45 A g^{-1})	328.8	91.2 (185)	91.7$_{10000\ cycles}$	[20]
Co_3O_4@NiO//Zn@Cu foil	6 M KOH	1.72	183(5 mA cm^{-2})	316.1	5.1 (130)	89.0 $_{500\ cycles}$	[21]
NNA@CNH//NNA@Zn	1 M KOH	1.75	246(5.0 A g^{-1})	148.5	13.8 (76)	88.0 $_{5000\ cycles}$	[22]
NiO/CNTs//Zn plate	1 M KOH + 10 mM $ZnAc_2$	1.75	155(1.0 A g^{-1})	228.3	4.4 (129)	65.0 $_{500\ cycles}$	[23]
CC-CCH@CMO//CC-ZnO@C-Zn	6 M KOH+1.5 M ZnO	1.65	0.7 mAh cm^{-2} (1 mA cm^{-2})	235.6	12.6 (152)	71.1 $_{5000\ cycles}$	[24]
CC-CF@NiO//CC-CF @ZnO	2 M KOH + sat. ZnO	1.75	203(0.5mA cm^{-2})	355.7	17.9 (210)	72.9 $_{2400\ cycles}$	[25]

Co_3O_4@NF//Zn @CF	1 M KOH + 10 mM $ZnAc_2$	1.78	150(3.0 A g^{-1})	241	14.0 (110)	80.0 $_{2000\ cycles}$	[26]

*E*mid,d is the middle discharge voltage. *W*max and *P*max represent the maximum energy density and power density, respectively. The data in the parenthesis is the corresponding energy density at the maximum power density.

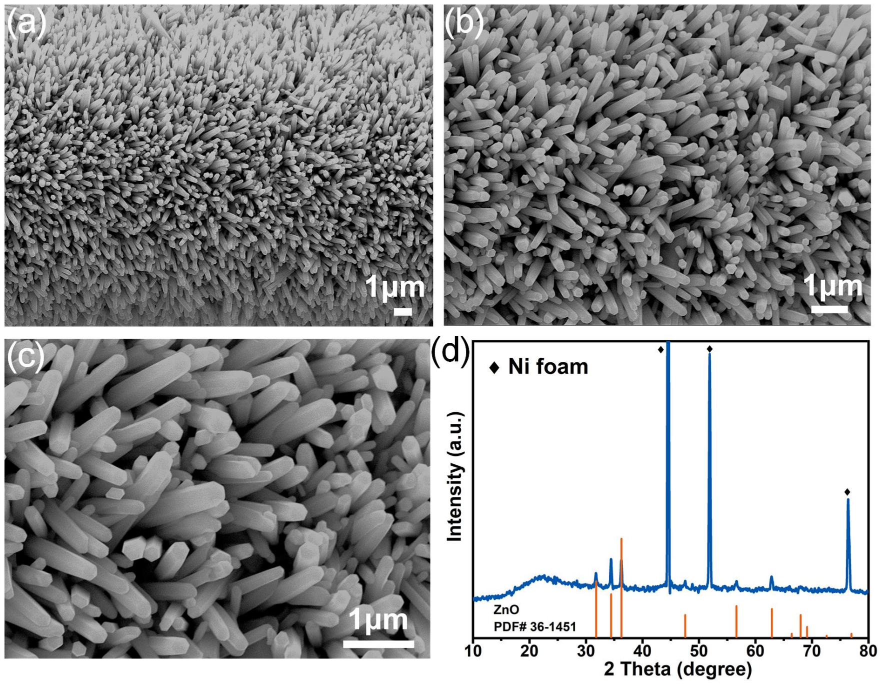

Fig. S1. a, b, c SEM of ZnO Nanorod Arrays (ZnO NAs) at different magnifications. **d** XRD patterns of ZnO NAs.

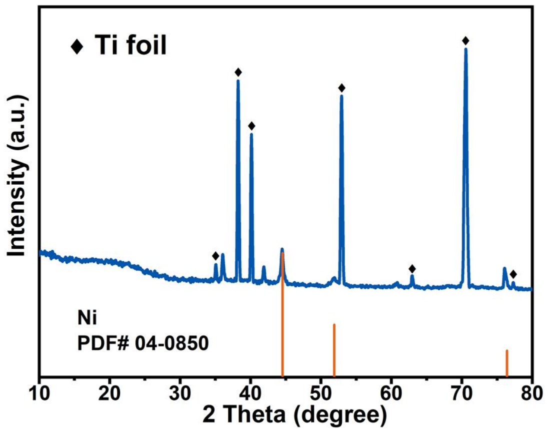

Fig. S2. XRD patterns of Ni NTs.

Here, in order to prevent the influence of foam nickel substrate on the test, we use Ti sheet as the electrodeposition substrate to prove that the electrodeposition product is nickel elemental.

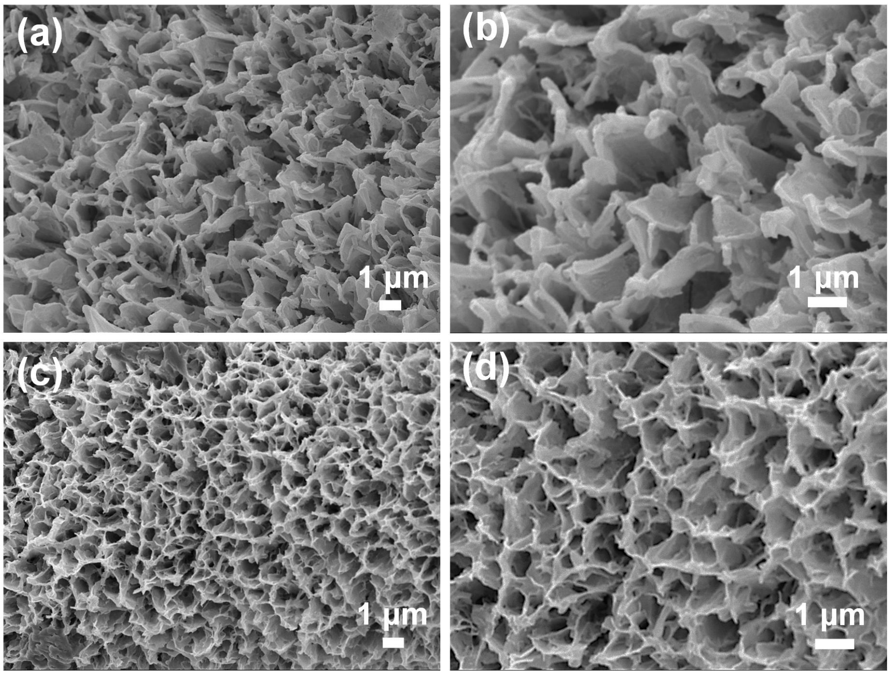

Fig. S3. a, b SEM images of CNO at different magnifications. **c, d** SEM images of O_d-CNO at different magnifications.

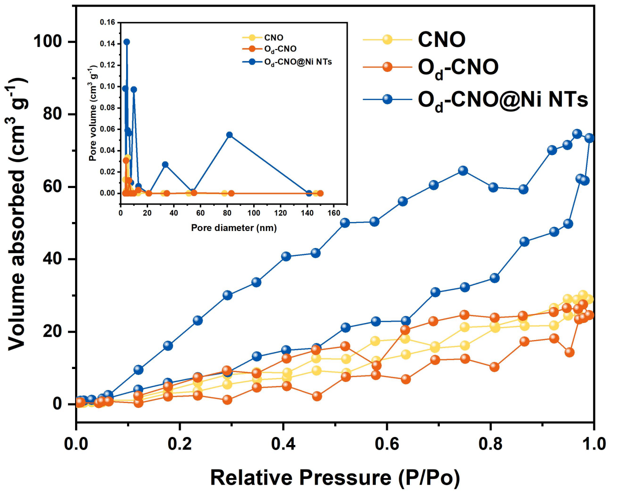

Fig. S4. Nitrogen adsorption–desorption isotherms and pore size distributions of O_d-CNO@Ni NTs, O_d-CNO and CNO

Here, since our sample is an array structure grown in situ on the nickel foam, the Brunauer-Emmett-Teller (BET) test results include the quality of the Ni foam.

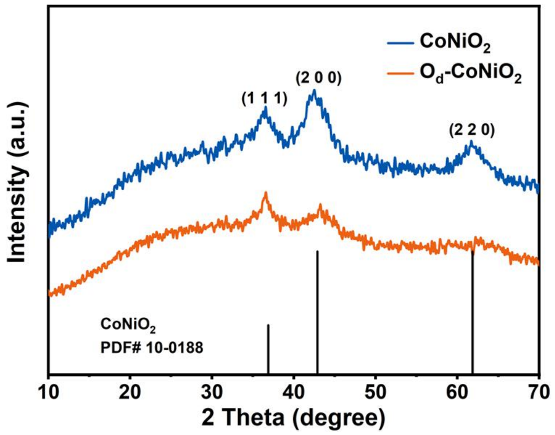

Fig. S5. XRD patterns of CNO and O_d-CNO.

Here, in order to prevent the influence of nickel foam substrate on the test, we pump and filter the powder sample for testing.

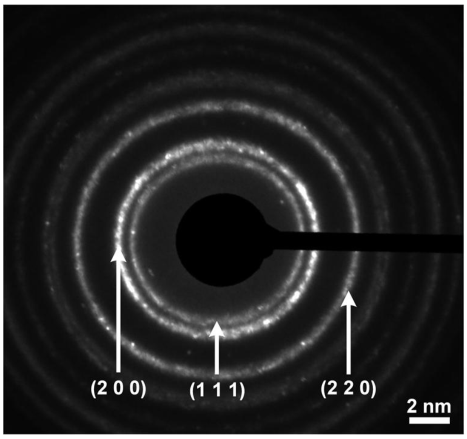

Fig. S6. The SAED pattern for the CNO sample.

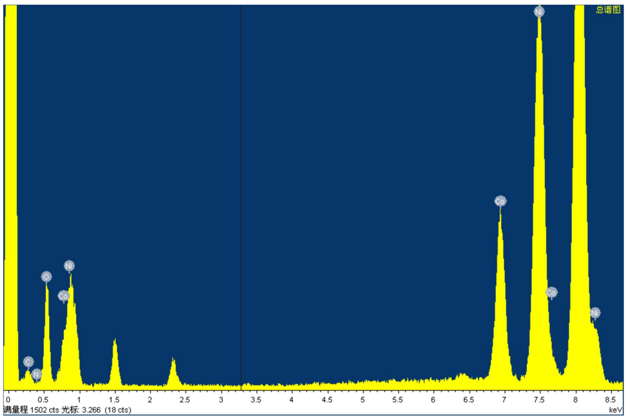

Fig. S7. Energy dispersive spectrum (EDS) of the O_d-CNO@Ni NTs sample.

Table 2. EDX results in elements content from the area identified in TEM micrograph (**Fig 2 e**).

Element	Spectral peak area	Area Sigma	K factor	wt%	wt% Sigma	Atomic percent
C K	406	68	2.208	1.70	0.28	5.94
N K	141	63	2.965	0.79	0.35	2.37
O K	3371	113	1.810	11.55	0.36	30.31
Co K	11533	205	1.239	27.06	0.43	19.27
Ni K	24982	298	1.245	58.90	0.54	42.11
**total**				100.00		

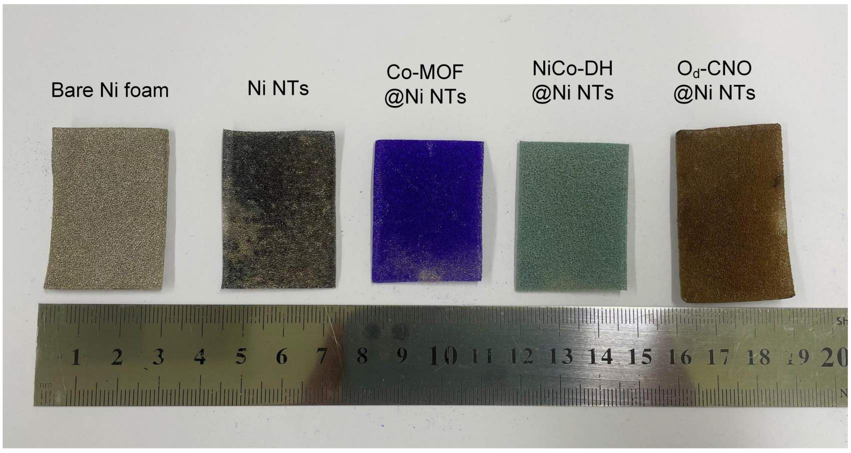

Fig. S8. Optical photograph of the material preparation process.

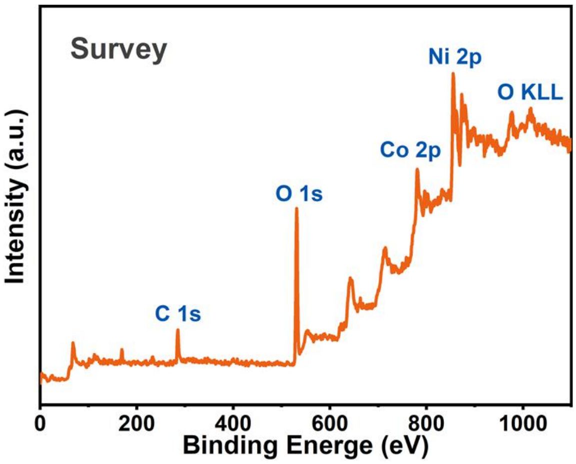

Fig. S9. XPS survey spectra of the O_d-CNO@Ni NTs.

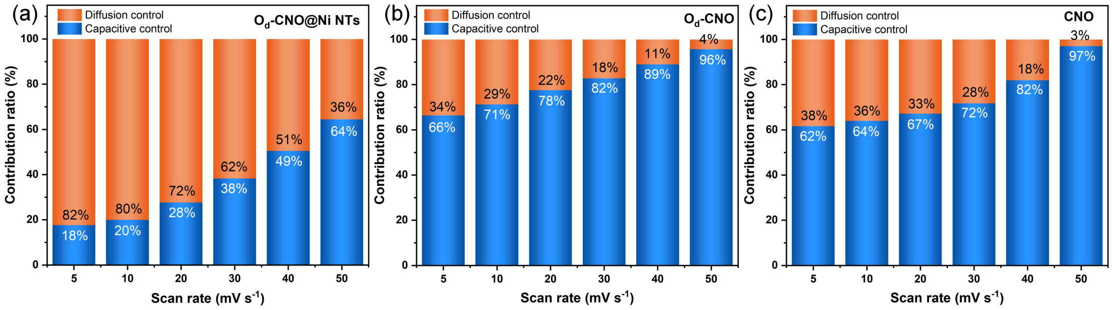

Fig. S10. capacity contribution ratio of **a** O_d-CNO@Ni NTs. **b** O_d-CNO. **c** CNO.

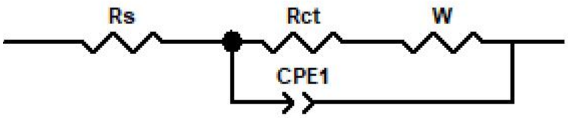

Fig. S11. EIS equivalent circuit diagram.

Table 3. The EIS results of CNO, O_d-CNO, O_d-CNO@Ni NTs.

	Rct	W
CNO	0.72 Ω	0.64 Ω
O_d-CNO	0.40 Ω	0.33 Ω
O_d-CNO@Ni NTs	0.35 Ω	0.07 Ω

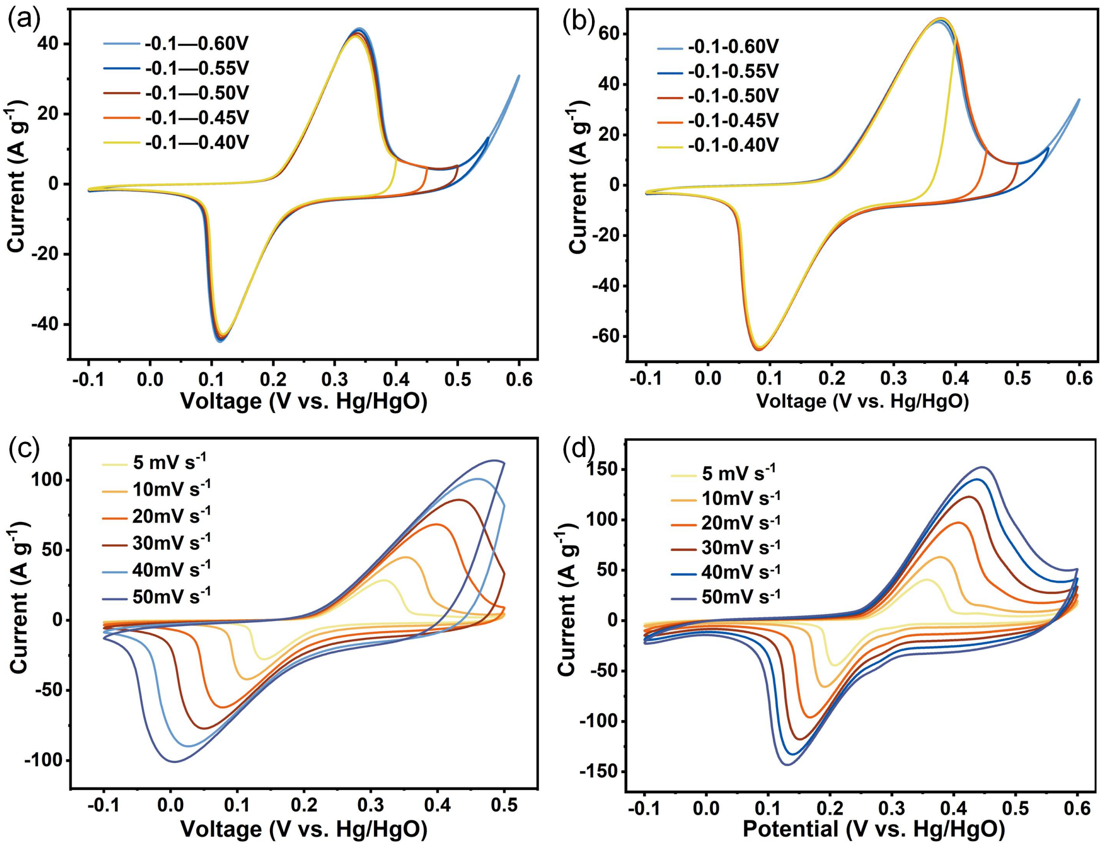

Fig. S12. The CNO cathode **a** The CV curve at different voltage Windows at 10 mV s^{-1}. **b** The CV curve at different voltage Windows at 20 mV s^{-1}. **c** CV curves. **d** CV curves of O_d-CNO cathodes.

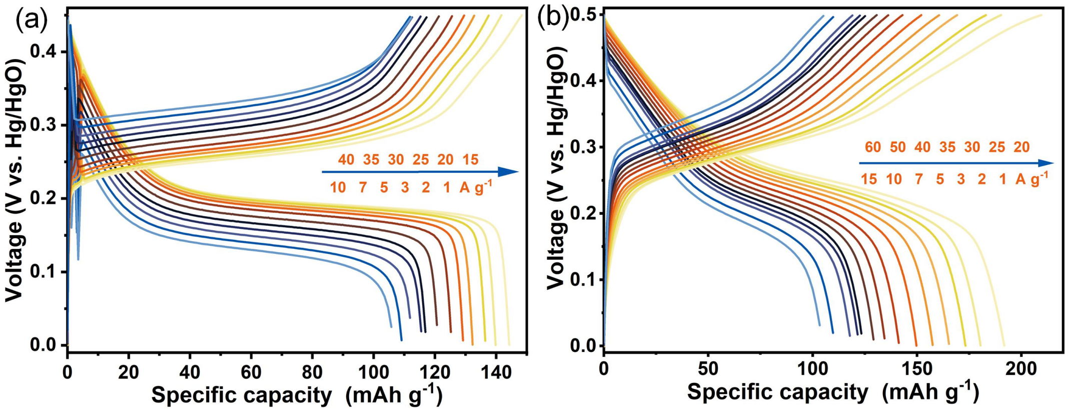

Fig. S13. **a** Galvanostatic charge-discharge curves of the CNO cathode. **b** Galvanostatic charge-discharge curves of the O_d-CNO cathode.

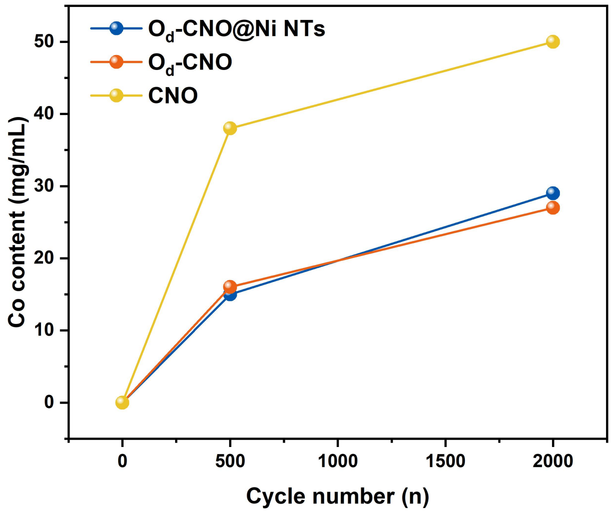

Fig. S14. Element analysis of dissolved Co ions in electrolyte during cycling by ICP-OES.

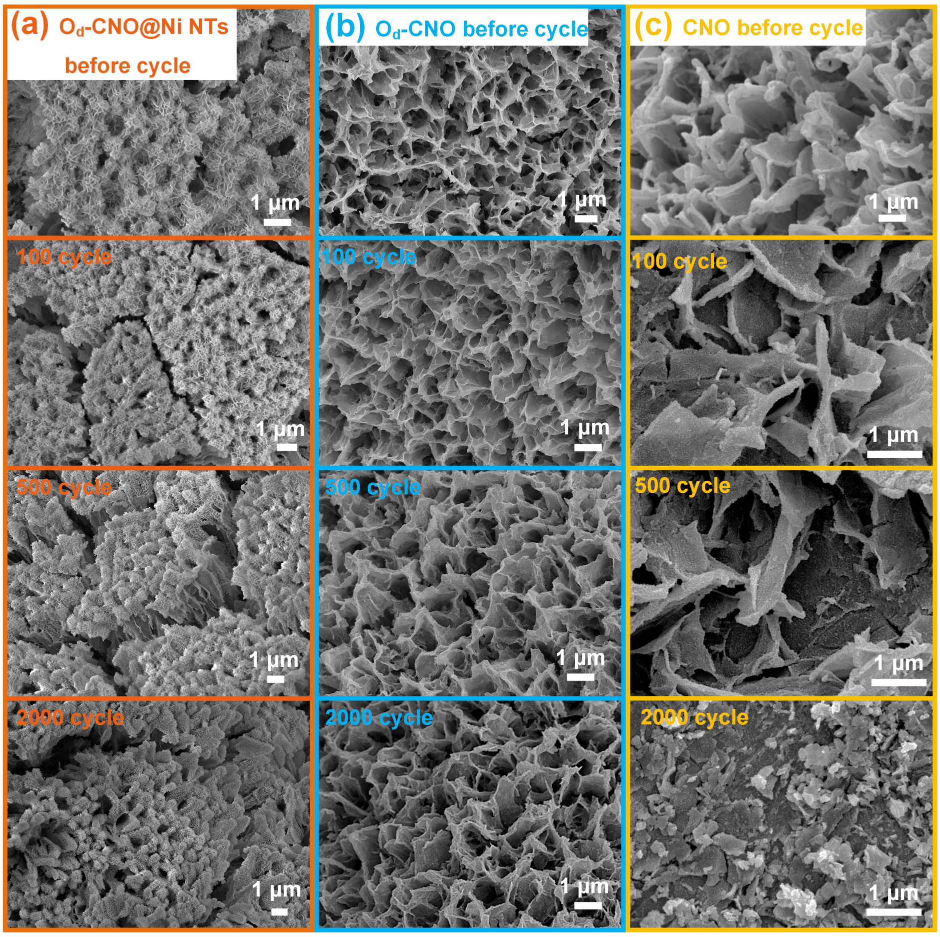

Fig. S15. The SEM images of O_d-CNO@Ni NTs, O_d-CNO and CNO electrodes with different number of cycles

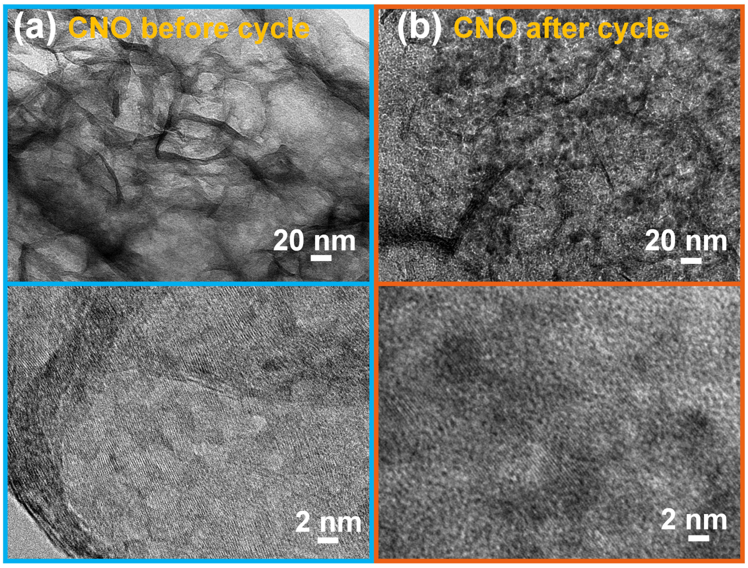

Fig. S16. The TEM images of CNO electrodes before and after cycles

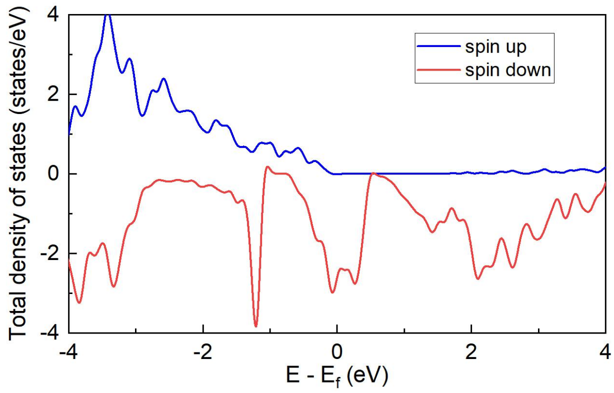

Fig. S17. The total density of states of CNO bulk.

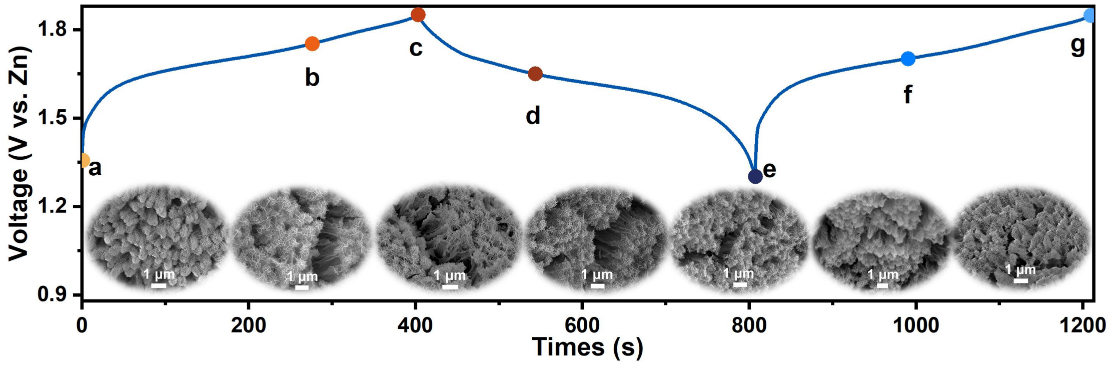

Fig. S18. Ex-situ SEM images at difffferent states.

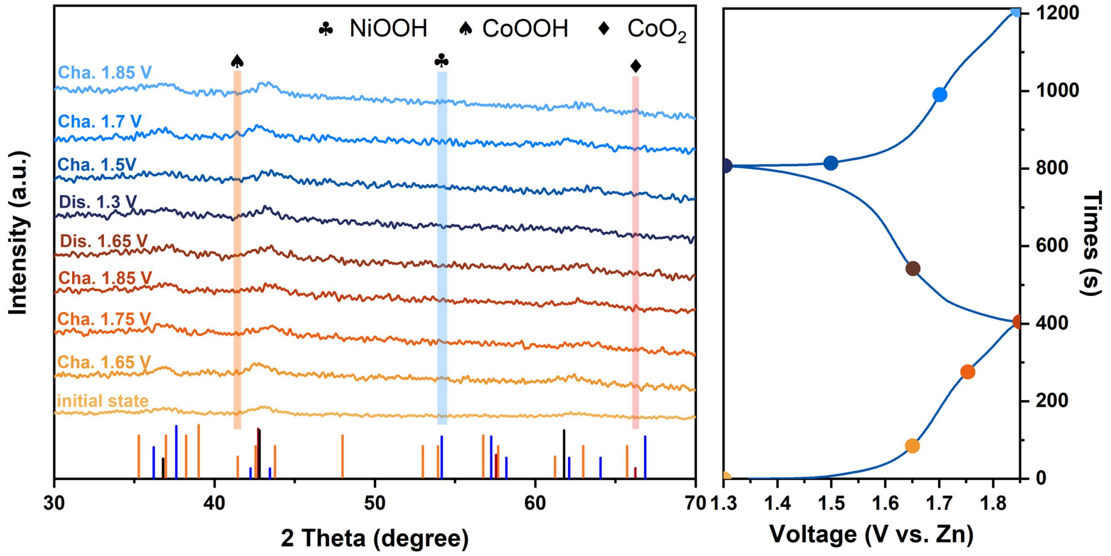

Fig. S19. Ex-situ XRD patterns at difffferent states.

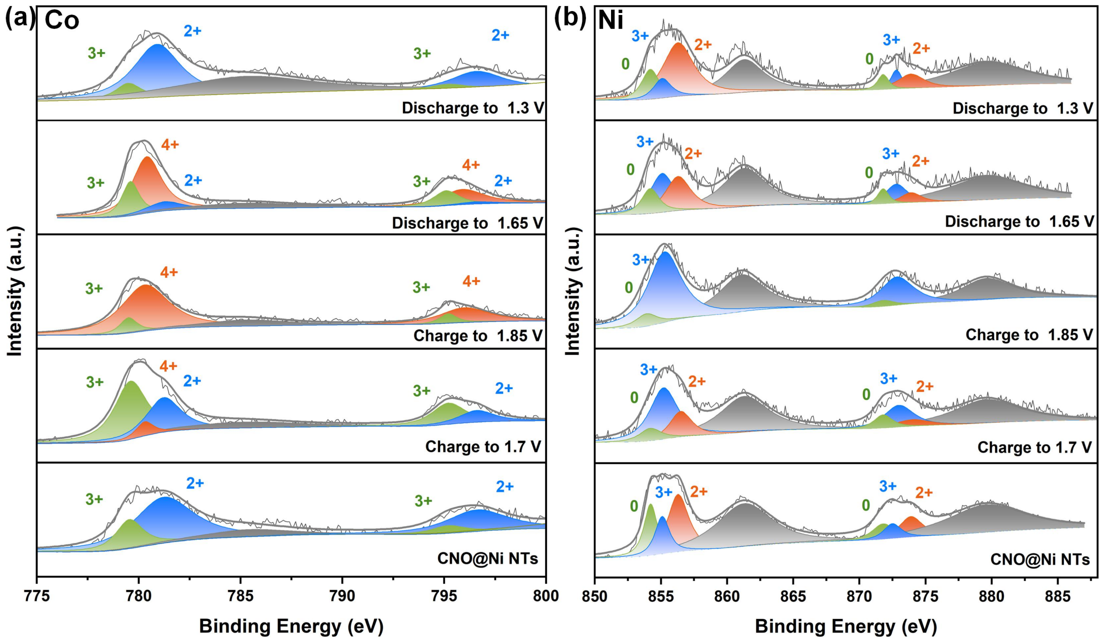

Fig. S20. Ex-situ XPS data at difffferent states.

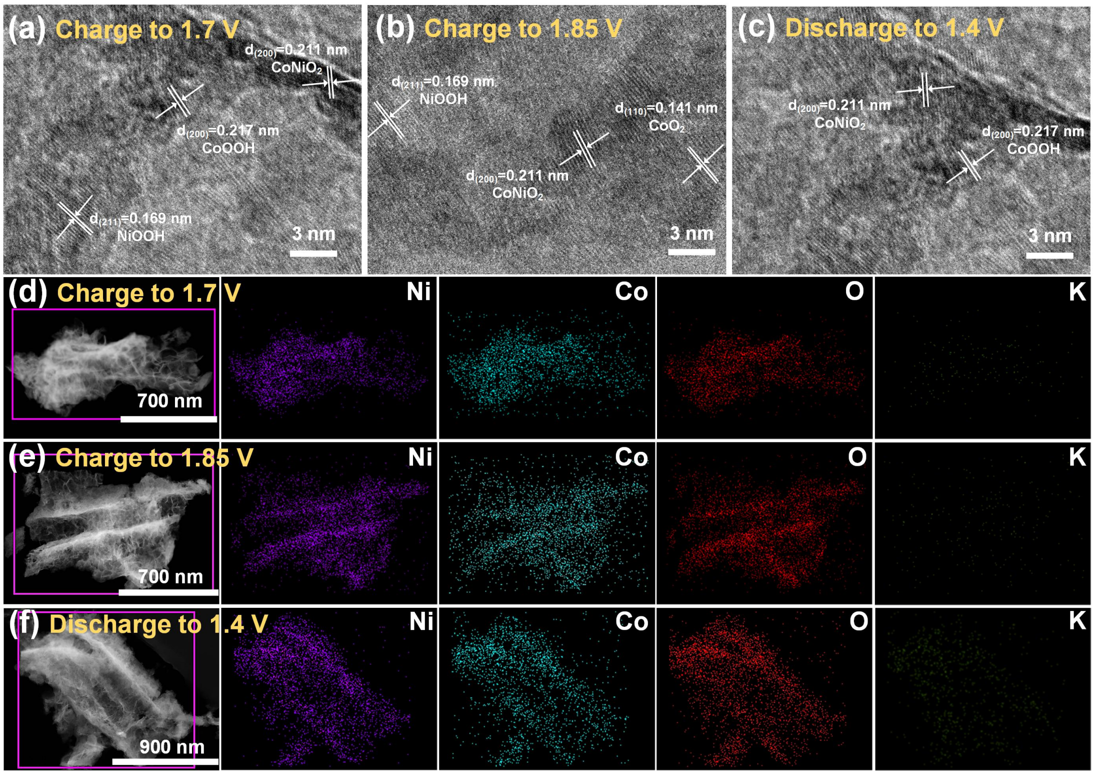

Fig. S21. a, b, c Ex-situ HRTEM images at difffferent states. **d, e, f** EDX element mappings at difffferent states.

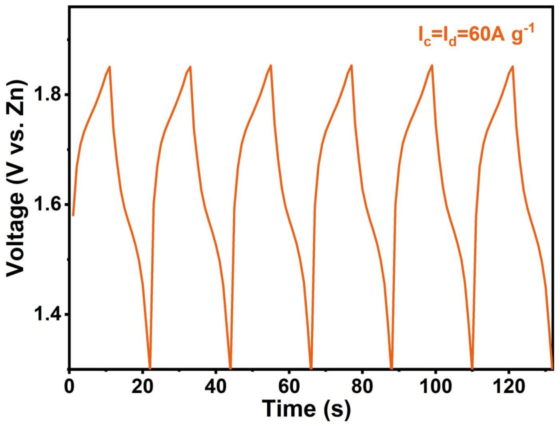

Fig. S22. The time *vs.* voltage curve of the O_d-CNO@Ni NTs//Zn aqueous battery at 60 A g^{-1}.

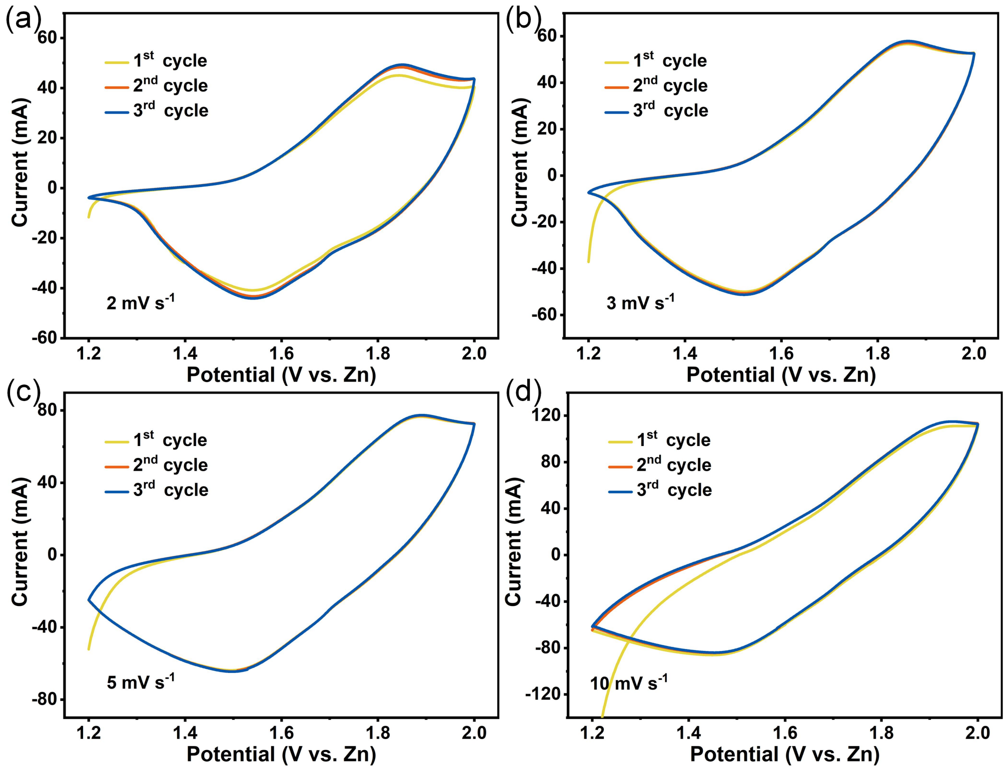

Fig. S23. Comparison of the CV curves of **a** 2 mV s^{-1} **b** 3 mV s^{-1} **c** 5mV s^{-1} **d**10 mV s^{-1}.

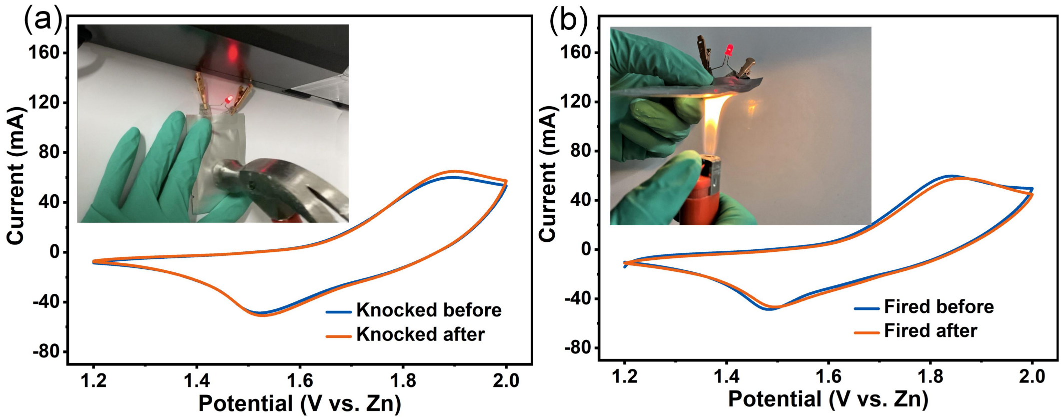

Fig. S24. Comparison of the CV curves before and after **a** fire and **b** knock.

References

[1] L. Li, L. Jiang, Y. Qing, Y. Zeng, Z. Zhang, L. Xiao, X. Lu, Y. Wu, Manipulating nickel oxides in naturally derived cellulose nanofiber networks as robust cathodes for high-performance Ni–Zn batteries. J. Mater. Chem. A 8 (2020) 565-572. https://doi.org/10.1039/C9TA09006A

[2] Y. Zeng, Z. Lai, Y. Han, H. Zhang, S. Xie, X. Lu, Oxygen-vacancy and surface modulation of ultrathin nickel cobaltite nanosheets as a high-energy cathode for advanced Zn-ion batteries. Adv. Mater. (2018) 1802396. https://doi.org/10.1002/adma.201802396

[3] S.W. Zhang, B.S. Yin, Y.Z. Luo, L. Shen, B.S. Tang, Z. Kou, X. Liu, D.B.K. Lim, D.M. Gu, Z.B. Wang, H. Gong, Fabrication and theoretical investigation of cobaltosic sulfide nanosheets for flexible aqueous Zn/Co batteries. Nano Energy 68 (2019) 104314. https://doi.org/10.1016/j.nanoen.2019.104314

[4] W. Zhou, D. Zhu, J. He, J. Li, H. Chen, Y. Chen, D. Chao, A scalable top-down strategy toward practical metrics of Ni–Zn aqueous batteries with total energy densities of 165 Wh kg^{-1} and 506 Wh L^{-1}. Energy Environ. Sci. 13 (2020) 4157-4167. https://doi.org/10.1039/D0EE01221A

[5] L. Zhou, X. Zhang, D. Zheng, W. Xu, J. Liu, X. Lu, Ni_3S_2@PANI core–shell nanosheets as a durable and high-energy binder-free cathode for aqueous rechargeable nickel–zinc batteries. J. Mater. Chem. A 7 (2019) 10629-10635. https://doi.org/10.1039/C9TA00681H

[6] W. Shang, W. Yu, P. Tan, B. Chen, H. Xu, M. Ni, A high-performance Zn battery based on self-assembled nanostructured $NiCo_2O_4$ electrode. J. Power Sources 421 (2019) 6-13. https://doi.org/10.1016/j.jpowsour.2019.02.097

[7] J. Wen, Z. Feng, H. Liu, T. Chen, Y. Yang, S. Li, S. Sheng, G. Fang, In-situ synthesized Ni_2P nanosheet arrays as the cathode for novel alkaline Ni//Zn rechargeable battery. Appl. Surf. Sci. 485 (2019) 462-467. https://doi.org/10.1016/j.apsusc.2019.04.222

[8] C. Teng, F. Yang, M. Sun, K. Yin, Q. Huang, G. Fu, C. Zhang, X. Lu, J. Jiang, Structural and defect engineering of cobaltosic oxide nanoarchitectures as an ultrahigh energy density and super durable cathode for Zn-based batteries. Chem. Sci. 10 (2019) 7600-7609. https://doi.org/10.1039/C9SC01902B

[9] H. Zhang, X. Zhang, H. Li, Y. Zhang, Y. Zeng, Y. Tong, P. Zhang, X. Lu, Flexible rechargeable Ni//Zn battery based on self-supported $NiCo_2O_4$ nanosheets with high power density and good cycling stability. Green Energy Environ. 3 (2018) 56-62. https://doi.org/10.1016/j.gee.2017.09.003

[10] H. Chen, Z. Shen, Z. Pan, Z. Kou, X. Liu, H. Zhang, Q. Gu, C. Guan, J. Wang, Hierarchical micro-nano sheet arrays of nickel-cobalt double hydroxides for high-rate Ni-Zn batteries. Adv. Sci. 6 (2019) 1802002. https://doi.org/10.1002/advs.201802002

[11] D. Amaranatha Reddy, H. Lee, M. Gopannagari, D. Praveen Kumar, K. Kwon, H.D. Yoo, T.K. Kim, Facile synthesis of cauliflower-like cobalt-doped Ni_3Se_2 nanostructures as high-performance cathode materials for aqueous zinc-ion

batteries. Int. J. Hydrogen Energy 45 (2020) 7741-7750. https://doi.org/10.1016/j.ijhydene.2019.06.004

[12] M. Cui, X. Bai, J. Zhu, C. Han, Y. Huang, L. Kang, C. Zhi, H. Li, Electrochemically induced $NiCoSe_2$@NiOOH/COOH heterostructures as multifunctional cathode materials for flexible hybrid Zn batteries. Energy Storage Mater. 36 (2021) 427-434. https://doi.org/10.1016/j.ensm.2021.01.015

[13] W. Liu, Y. Chen, Y. Wang, Q. Zhao, L. Chen, W. Wei, J. Ma, Influence of anion substitution on 3d-architectured Ni-Co-A (A=H, O, P) as efficient cathode materials towards rechargeable Zn-based battery. Energy Storage Mater. 37 (2021) 336-344. https://doi.org/10.1016/j.ensm.2021.02.026

[14] X. Zhu, Y. Wu, Y. Lu, Y. Sun, Q. Wu, Y. Pang, Z. Shen, H. Chen, Aluminum-doping-based method for the improvement of the cycle life of cobalt-nickel hydroxides for nickel-zinc batteries. J. Colloid Interface Sci. 587 (2021) 693-702. https://doi.org/10.1016/j.jcis.2020.11.029

[15] X. Wang, Z. Yang, P. Zhang, Y. He, Z.A. Qiao, X. Zhai, H. Huang, $Ni(OH)_2$ cathode with oxygen vacancies induced from electroxidizing Ni_3S_2 nanosheets for aqueous rechargeable Ni–Zn battery. J. Alloys Compd. 855 (2021) 157488. https://doi.org/10.1016/j.jallcom.2020.157488

[16] Y. Shen, K. Zhang, F. Yang, Z. Li, Z. Cui, R. Zou, Q. Liu, J. Hu, K. Xu, Oxygen vacancies-rich cobalt-doped $NiMoO_4$ nanosheets for high energy density and stable aqueous Ni-Zn battery. Sci. China Mater. 63 (2020) 1205-1215. https://doi.org/10.1007/s40843-020-1292-6

[17] M. Gong, Y. Li, H. Zhang, B. Zhang, W. Zhou, J. Feng, H. Wang, Y. Liang, Z. Fan, J. Liu, H. Dai, Ultrafast high-capacity NiZn battery with NiAlCo-layered double hydroxide. Energy Environ. Sci. 7 (2014) 2025-2032. https://doi.org/10.1039/C4EE00317A

[18] Y. Jian, D. Wang, M. Huang, H.-L. Jia, J. Sun, X. Song, M. Guan, Facile synthesis of $Ni(OH)_2$/carbon nanofiber composites for improving NiZn battery cycling life. ACS Sustain. Chem. Eng. 5 (2017) 6827-6834. https://doi.org/10.1021/acssuschemeng.7b01048

[19] P. Hu, T. Wang, J. Zhao, C. Zhang, J. Ma, H. Du, X. Wang, G. Cui, Ultrafast alkaline Ni/Zn battery based on Ni-foam-supported Ni_3S_2 nanosheets. ACS Appl. Mater. Interfaces 7 (2015) 26396-26399. https://doi.org/10.1021/acsami.5b09728

[20] W. Zhou, J. He, D. Zhu, J. Li, Y. Chen, Hierarchical $NiSe_2$ nanosheet arrays as a robust cathode toward superdurable and ultrafast Ni-Zn aqueous batteries. ACS Appl. Mater. Interfaces 12 (2020) 34931-34940. https://doi.org/10.1021/acsami.0c08205

[21] Z. Lu, X. Wu, X. Lei, Y. Li, X. Sun, Hierarchical nanoarray materials for advanced nickel–zinc batteries. Inorg. Chem. Front. 2 (2015) 184-187. https://doi.org/10.1039/C4QI00143E

[22] C. Xu, J. Liao, C. Yang, R. Wang, D. Wu, P. Zou, Z. Lin, B. Li, F. Kang, C.P. Wong, An ultrafast, high capacity and superior longevity Ni/Zn battery constructed on nickel nanowire array film. Nano Energy 30 (2016) 900-908. https://doi.org/10.1016/j.nanoen.2016.07.035

[23] X. Wang, M. Li, Y. Wang, B. Chen, Y. Zhu, Y. Wu, A Zn–NiO rechargeable battery with long lifespan and high energy density. J. Mater. Chem. A 3 (2015) 8280-8283. https://doi.org/10.1039/C5TA01947H

[24] M. Li, J. Meng, Q. Li, M. Huang, X. Liu, K.A. Owusu, Z. Liu, L. Mai, Finely crafted 3D electrodes for dendrite-free and high-performance flexible fiber-shaped Zn-Co batteries. Adv. Funct. Mater. 28 (2018) 1802016. https://doi.org/10.1002/adfm.201802016

[25] J. Liu, C. Guan, C. Zhou, Z. Fan, Q. Ke, G. Zhang, C. Liu, J. Wang, A flexible quasi-solid-state nickel-zinc battery with high energy and power densities based on 3D electrode design. Adv. Mater. 28 (2016) 8732-8739. https://doi.org/10.1002/adma.201603038

[26] X. Wang, F. Wang, L. Wang, M. Li, Y. Wang, B. Chen, Y. Zhu, L. Fu, L. Zha, L. Zhang, Y. Wu, W. Huang, An aqueous rechargeable Zn//Co_3O_4 battery with high energy density and good cycling behavior. Adv. Mater. 28 (2016) 4904-4911. https://doi.org/10.1002/adma.201505370
